# Experimental Characterization and Numerical Modeling of the Corrosion Effect on the Mechanical Properties of the Biodegradable Magnesium Alloy WE43 for Orthopedic Applications

**DOI:** 10.3390/ma15207164

**Published:** 2022-10-14

**Authors:** Felipe Saconi, Geraldine Hincapie Diaz, André Costa Vieira, Marcelo Leite Ribeiro

**Affiliations:** 1 Aeronautical Engineering Department, São Carlos School of Engineering, University of São Paulo, São Carlos 13563-500, SP, Brazil; 2 Center for Mechanical and Aerospace Science and Technologies (C-MAST-UBI), Universidade da Beira Interior, R. Marquês D’Ávila e Bolama, 6201-001 Covilhã, Portugal

**Keywords:** biodegradable magnesium alloy WE43, numerical simulation, continuum damage mechanics, corrosion, finite elements method

## Abstract

Computational modeling plays an important role in the design of orthopedic implants. In the case of biodegradable magnesium alloys, a modeling approach is required to predict the effects of degradation on the implant’s capacity to provide the desired stabilization of fractured bones. In the present work, a numerical corrosion model is implemented to predict the effects of biodegradation on the structural integrity of temporary trauma implants. A non-local average pitting corrosion model is calibrated based on experimental data collected from in vitro degradation experiments and mechanical testing of magnesium WE43 alloy specimens at different degradation stages. The localized corrosion (pitting) model was implemented by developing a user material subroutine (VUMAT) with the program Abaqus^®^/Explicit. In order to accurately capture both the linear mechanical reduction in specimen resistance, as well as the non-linear corrosion behavior of magnesium WE43 observed experimentally, the corrosion model was extended by employing a variable corrosion kinetic parameter, which is time-dependent. The corrosion model was applied to a validated case study involving the pull-out test of orthopedic screws and was able to capture the expected loss of screw pull-out force due to corrosion. The proposed numerical model proved to be an efficient tool in the evaluation of the structural integrity of biodegradable magnesium alloys and bone-implant assembly and can be used in future works in the design optimization and pre-validation of orthopedic implants.

## 1. Introduction

The use of temporary implants for osteosynthesis, such as orthopedic nails, plates and screws, is a well-accepted technique for stabilizing fractures. These devices provide anatomic bone alignment and stabilization, reducing the malunion incidence and fragmentary nonunion, and allow the early mobilization of the patient [1]. Currently, titanium alloys and stainless steels are the most used biomaterials to manufacture implants for osteosynthesis, with titanium representing 45.1% of the market in 2017 [2]. However, some negative aspects are associated with the use of these biomaterials: stress shielding and resorption of newly healed bone, stress concentration in the fixation device and device failure (e.g., plate cracking or screw pull-out), inflammation, post-surgery infection, interference in radiological exams, among others [3,4,5].

In many cases, these negative aspects lead to the need of a second surgery for implant removal [5]. Although these are relatively simple procedures, explant surgeries have a large impact on patient health—which is again exposed to the several risks of a surgical procedure—and on the economic character, due to the associated surgical costs and the post-operative recovery time [6].

In the last decade, advances have been made in the development of biodegradable metallic materials, most pronouncedly in magnesium alloys. Through the addition of alloying elements, surface modifications and improvements in processing methods, it was possible to solve the problem of the high rate of biodegradation of magnesium in vivo, thus, culminating in the commercialization of the first implants manufactured with this class of materials [7,8,9]. Magnesium alloys have several benefits when compared to current bioinert metallic materials. They are bioabsorbable and biocompatible, less dense and have a similar stiffness to bone tissue, thus, minimizing the stress-shielding issue [4,5,10].

Therefore, bearing in mind that a deep understanding of the mechanisms of magnesium degradation in vivo and its effects on the mechanical properties of materials is of great importance for the popularization of its use, several types of research have sought to characterize the mechanisms and rates of the degradation of magnesium alloys in vivo and in corrosive environments similar to the physiological study (in vitro), and to simulate them in silico through finite element analysis [11,12,13,14].

For this reason, this study covers one of the most important corrosion mechanisms. Pitting corrosion is a localized form of corrosion, where cavities or “holes” are produced in the material. Pitting is considered more dangerous than uniform corrosion damage since it is more difficult to detect, predict and design [4,8,15,16]. The localized corrosion mechanisms are generally caused by impurities and inhomogeneities in the material, which are largely unavoidable due to the manufacturing process of such alloys. While the spatial and temporal evolution of corrosion can be controlled to some degree by varying the alloying composition and/or by applying a surface coating [8,9,10,11,12], it is generally not possible to achieve uniform or non-localized corrosion in magnesium-based biomaterials [16].

**Figure 1:**
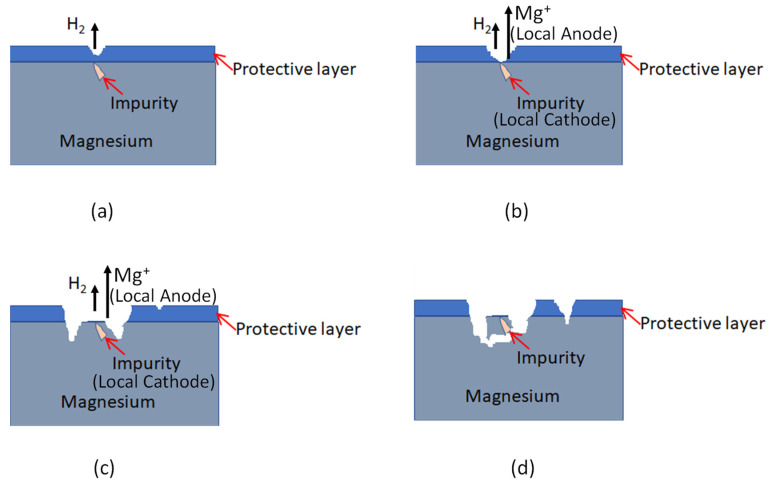
The localized corrosion mechanism of magnesium in aqueous media; (**a**) corrosion in regions with cracks in the passive layer or the vicinity of impurities; (**b**) there is an increase in immersion time, the passive layer is removed in a localized region, resulting in the formation of local anodes and cathodes on the surface; (**c**) after a long period of immersion; (**d**) a high degree of localized corrosion occurs, with additional mass loss due to the detachment of particles due to erosion.

Immediately after immersion, a protective oxide film covers the magnesium surface. Then, corrosion will occur in the vicinity of impurities. Over time, the film is removed in a localized region and local cathodes and anodes can form on the surface. After a longer period, a large degree of localized corrosion occurs. Finally, we have mass loss due to particle undermining. The localized corrosion mechanism of magnesium in aqueous media is illustrated in Figure 1. For short immersion periods and for low potential differences, the passive layer covers the surfaces of the material and is able to protect it from corrosion [15,16].

Some studies provide limited qualitative assessments of surface corrosion through visual examination and there is a lack of quantitative data on the spatial progression of surface corrosion. Furthermore, only a limited number of studies provide quantitative results (pit depth, pit density and pitting factor) regarding the severity of localized corrosion for Mg alloys. Few in vivo and in vitro studies have quantified the non-uniform relationship between specimen corrosion and mechanical strength of magnesium alloys undergoing corrosion [16]. The disproportionate reduction in load-bearing capacity, compared to corresponding mass loss, is clearly attributed to pitting corrosion.

In general, finite element analysis has been recognized as a powerful tool in orthopedic product development. Using “Digital Twin” is easier and faster to optimize the final shape of the implant according to design criteria, such as strength or stiffness. The main use of computational analysis occurs in the evaluation of the performance of devices in the initial stages of development, allowing improvements and optimizations in the design before the construction of prototypes, resulting in greater agility to virtually test different geometries, thus, reducing design costs and the number of mechanical and in vivo tests to evaluate or pre-validate the implant degradation behavior. Among the phenomenological or physical approaches discussed, Abdalla et al. [4] stated that in the literature, different authors have used a phenomenological-based continuum damage mechanics (CDM) approach for predicting the performance of biodegradable materials. Gastaldi et al. [11] developed a multi-mechanism corrosion model within the framework of CDM to simulate both uniform and stress corrosions of magnesium alloys for coronary stent applications. Grogan et al. [12] extended the uniform corrosion model proposed by Gastaldi et al. [11] to account for the localized pitting corrosion mechanism present in most biodegradable magnesium alloys. As these models are phenomenological-based, they evaluate the material corrosion damage by the means of arbitrary state variables, which progressively degrade the mesh elements without explicitly modeling the corrosion phenomena and their evolution. In other words, they do not capture physical and chemical processes that are responsible for causing the corrosion, such as electrochemical surface reactions [4,11,12].

The main objective of this work was to implement a finite element model capable of simulating the biodegradation of magnesium alloy WE43 under conditions that are similar to those of the human body, and capturing its long-term effects on mechanical properties in order to assist in the mechanical design of biodegradable implants, for example, pins, screws and plates. For this purpose, the magnesium alloy WE43 was mechanically characterized by tensile testing, enabling the calibration of the finite element model for non-degraded material. Furthermore, the magnesium alloy WE43 was mechanically characterized after different stages of degradation in a simulated body fluid. Hence, it was possible to analyze the influence on the material structural integrity for continuum damage model calibration purposes. Finally, the pull-out force evolution along screw corrosion was simulated, using a case study of bone-screw assembly.

## 2. Materials and Methods

### 2.1. Magnesium Alloy WE43MEO

Magnesium alloy WE43MEO (Meotec GmbH, Aachen, Germany) with a nominal composition of 1.4–4.2% Y, 2.5–3.5% Nd, <1% (Al, Fe, Cu, Ni, Mn, Zn, Zr) and balance Mg (in wt%) was chosen to carry out the studies, since this class of alloys are potential candidates for future bioabsorbable orthopedic implant materials due to their biocompatibility, mechanical properties similar to human bone, and the ability to completely degrade in vivo [17]. It is currently being used in the production of orthopedic screws. In addition, the WE43MEO alloy was specially developed for use as a biodegradable material, with a refined microstructure and low level of impurities. The material was supplied in a wire form, with a diameter of 0.84 mm and in the cold-worked condition (TF) at 40%, where the TF is the percentual area reduction.

### 2.2. Mechanical Characterization

To evaluate the mechanical properties of magnesium alloy WE43MEO, a uniaxial tensile test was carried out to evaluate the following mechanical properties: modulus of elasticity, yield strength and the ultimate tensile strength (UTS). The material was tested in two different thermomechanical conditions: as-drawn, with no thermal treatment, and annealed at 400 °C for 15 min [18]. Non-standardized specimens (CP) with total dimensions of Ø0.84 mm × 84 mm and a useful length of 50 mm were used. An MTS Bionix Servohydraulic Test System tensile test machine, with a 15 kN load cell, was used for mechanical characterization. The tensile tests were conducted at a constant displacement rate of 1 mm/min. Data were collected using the TestWorks 4 at 1 Hz frequency and were analyzed using the Minitab 17 software.

### 2.3. In Vitro Biodegradation Test

Corrosion experiments were developed through in vitro immersion to characterize the biodegradation of the magnesium alloy WE43MEO. A first experiment was performed to determine the corrosion rate of the material when immersed in Simulated Body Fluid (SBF) using the hydrogen evolution method. A second test was carried out to determine the corrosion effect on the material mechanical properties through immersion in SBF followed by a tensile test [15].

For the in vitro biodegradation test, the electrolyte used was the conventional simulated body fluid (c-SBF) due to its great popularity of use and good ionic and pH stability for storage [15]. The SBF was produced according to the methodology proposed by Oyane et al. [19]. All in vitro biodegradation tests were performed at 37 ± 0.5 °C, controlled through a microcontroller heating system. In addition, the pH was controlled daily and maintained at values close to physiological values, in the range of 7.4 to 8. The preparation of the specimens before measuring their final weight consisted of chemical cleaning with 20% chromic acid, followed by cleaning in an ultrasonic bath with ethanol and distilled water and drying in oven at 50 °C.

### 2.4. Determining the Rate of Biodegradation

**Figure 2:**
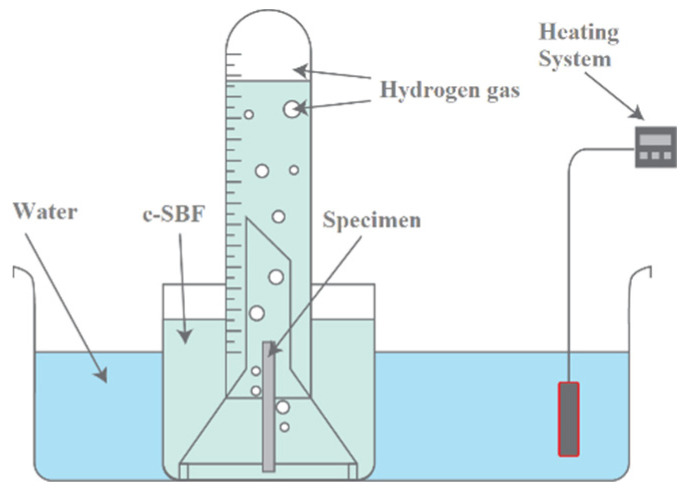
Hydrogen gas evolution method set up.

The determination of the WE43MEO magnesium biocorrosion rate was performed using the hydrogen evolution measurement method, originally proposed by Song, Atrens, and Stjohn [20], which uses a system consisting of a container, funnel and a 15 mL graduatedtube with a resolution of 0.2 mL, all made of glass, as shown in Figure 2 [21].

For this test, specimens measuring Ø0.84 mm × 30 mm were used—they had a total surface area of 0.7917 cm^2^ and an electrolyte volume of approximately 350 mL, resulting in a ratio of 442 mL/cm^2^, which is higher than the critical volume of 6.7 mL/cm^2^ [21]. The hydrogen volume released was measured with the following periodicity: 0.5 h, 1 h, 3 h, 6 h, 12 h, 24 h, 48 h, 72 h and 168 h.

### 2.5. Biodegradation Effect on Mechanical Properties

The corrosion effect on the material mechanical properties was analyzed through immersion in SBF during different stages, followed by tensile testing. After preparation, every specimen was identified, and their mass was measured using a precision scale with a resolution of 0.1 mg. In addition, on both ends of the specimens, a protective layer of Polytetrafluoroethylene (PTFE) was applied along a length of 17 mm. The PTFE acts as a barrier between the material surface and the SBF, restricting the corrosion area to the 50 mm central part of the specimen. This way, we ensured that failure of the specimen during tensile testing would occur in the central region.

Therefore, the working area of the specimen was restricted to a length of 50 mm, resulting in a surface area subject to corrosion of 1.32 cm^2^. For each degradation stage, 3 specimens (n = 3) were used. The specimens used in these tests were removed from the bath at regular time intervals: 6 h, 12 h, 24 h, 48 h, 72 h and 168 h. At the end of each degradation stage, a cleaning process was carried out by immersing the specimens in a 20% chromic acid solution, followed by rinsing in distilled water to remove all possible corrosion products from the surface of the specimens. After this, specimens were finally tensile tested according to the procedure described above [21].

### 2.6. Implementation of the Biodegradation Model

The corrosion damage model was implemented through a subroutine written in Fortran^®^, called VUMAT–User Material Subroutine, which is compatible with the commercial finite element software Abaqus^®^/Explicit 6.14 (Dassault Systèmes, Providence, RI, USA). The use of a VUMAT together with Abaqus^®^/Explicit allows the user to define constitutive models for the continuum finite description of materials of different complexities and different from those already available in the material library of Abaqus^®^.

### 2.7. Mathematical Description of the Model

Localized corrosion (pitting) was implemented using a non-local integral formulation, aiming to reduce the influence of mesh size on the results. These problems are intrinsic to models based on continuous damage mechanics [4,16]. The von Mises elastoplastic constitutive model, with isotropic hardening, was used to model the WE43 magnesium alloy mechanical behavior. The model can simulate mass loss during the corrosion process, along a continuous solid body, as well as the effects of small-scale corrosion-induced defects on the mechanical integrity of the material—in this case, along the 3D external surface of the solid. In this model, the corroding environment is unable to diffuse into the material, and corrosion proceeds from the surface and in particular points. As corrosion proceeds, the formation of higher roughness at the surface will increase the current area of the initial surface area.

The localized corrosion damage evolution law, initially proposed by Grogan et al. [12] and extended by Ma et al. [22], in Equation (1), is expressed in the non-local integration formulation as:

(1) $$\frac{dD_{p}}{dt}=\lambda_{e}^{*}k_{p}$$

where $k_{p}$ is the corrosion kinetic parameter, which must be calibrated experimentally by inverse parameterization, and $\lambda_{e}^{*}$ is the non-local average pitting parameter of a material point, according to the position vector x, which is calculated by Equations (2) and (3):

(2) $$\lambda_{e}^{*}\left(x\right)=\int_{V}^{}\alpha \left(x,y\right)\lambda_{e}^{}\left(y\right)dV$$

where $\lambda_{e}$ is the local pitting parameter of a material point with the position vector *y* in the influencing zone of the material point *x*, and α(x,y) is the non-local average operator, as defined on the work of [23].

(3) $$\alpha \left(x,y\right)=\left(\frac{\alpha_{0}\left(x,y\right)}{\int_{V}^{}\alpha_{0}\left(x,y\right)dV}\right)$$

where $\alpha_{0}\left(x,y\right)$ is the weight function defined by Equation (4):

(4) $$\alpha_{0}\left(x,y\right)={\left(1-\frac{\left(x-y\right)\left(x-y\right)}{L_{r}{}^{2}}\right)}^{2}$$

where $L_{r}$ is a scalar related to the intrinsic length, used by Ma et al. [22], which is defined as the greatest possible distance from a material point influencing the pitting behavior of a material point x.

The value of the local pitting parameter $\lambda_{e}$ varies from 0 to 1. The Weibull distribution probability density function (PDF), of the pitting parameter $\lambda_{e}$ determines the heterogeneity of the corrosive process and follows a Weibull distribution [4,16]. In this way, the value of $\lambda_{e}$ is computed according to a Weibull distribution, given by Equation (5). The probability of the value of $\lambda_{e}$ varying from “*a*“ to “*b*“ is given by Equation (6).

(5) $$\left(x\;:\;\psi ,\;\;\;\gamma \right)=\{\begin{array}{c} \frac{\gamma }{\psi }\;{\left(\frac{x}{\psi }\right)}^{\gamma -1}e^{-{\left(x/\psi \right)}^{k}}\;\;\;\;\;x\geq 0\;\\ 0\;\;\;\;\;\;\;\;\;\;\;\;\;\;\;\;\;\;\;\;\;\;\;\;\;\;\;\;\;\;\;\;\;\;\;\;\;\;x<0 \end{array}$$

(6) $$\mathrm{Pr}\left[a\;\leq \;\lambda_{e}\;\leq b\right]=\int_{a}^{b}f\left(x\right)dx$$

where γ and ψ are dimensionless parameters of the Weibull distribution that determine the heterogeneity of localized corrosion and must be calibrated experimentally.

Corrosion is only allowed to occur on surface elements which are in direct contact with the corrosion environment. When the overall damage parameter D of one element reaches the critical value equal to 0.999, the element is removed from the FE mesh. After a damaged element is deleted from the FE mesh, the material surface morphology and mass loss of that hexahedral volumetric element are updated [22]. In order to maintain the pitting corrosion process within the simulation, as the material surface morphology is updated, the neighboring elements have their pitting parameter scaled, as proposed by [12], according to Equation (7):

(7) $$\lambda_{e}^{\prime }=\beta \;\lambda_{e},$$

where $\lambda_{e}^{\prime }$ is the scaled local pitting parameter of a neighboring element, $\lambda_{e}$ is the value of the local pitting parameter of the removed element and *β* is a dimensionless parameter that controls the pit growth within the FE analysis, which must be calibrated based on experimental data.

The corrosion model implemented in the present work consists of three components: a preprocessor, a VUMAT and a “DAMAGE” subroutine.

The preprocessor, which runs before the main analysis of Abaqus^®^, has the function of preparing the input file for the main analysis based on the Abaqus^®^ .inp file. This pre-processing step encompasses the acquisition of vital data for the execution of the model, such as:

- The generation of the element connectivity map, or identification of neighboring elements to each element. Neighboring elements are those that share a face between themselves.
- The generation of coordinate and volume maps.
- The determination of the elements located at the surface of the model.
- The assignment of initial local pitting parameters to each of the elements located at the initial surface of the model, according to the Weibull distribution.

**Figure 3:**
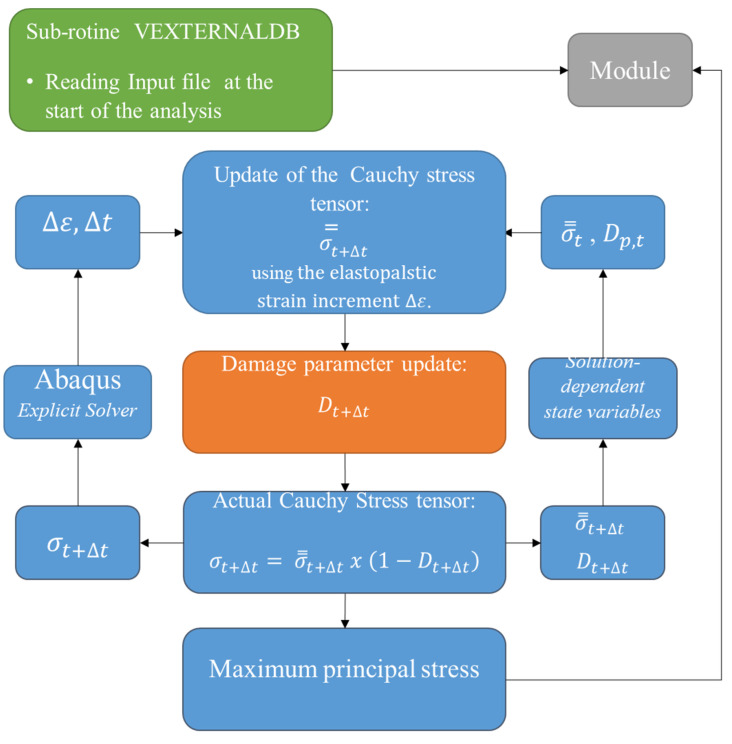
VUMAT operation flowchart.

The main analysis of Abaqus^®^/Explicit is performed through a VUMAT that describes the constitutive behavior of the magnesium alloy, coupled with a “DAMAGE” subroutine, which is used to update the value of the damage parameter *D*. This parameter modifies the constitutive behavior of the material to represent the effects of damage caused by corrosion. Figure 3 presents a flowchart of the VUMAT operation.

After starting the main analysis, the information contained in the input file is read through the VEXTERNALDB subroutine and saved in global variables through the MODULE function, present in FORTRAN 90. The VEXTERNALDB subroutine is called automatically by the Abaqus^®^ main analysis in different analysis stages: at the beginning and end of the analysis, and at the beginning and end of each increment of degradation time. At each of these call points, the subroutine allows, among other possibilities, the opening and closing of external files to exchange data with Abaqus^®^ analysis and the exchange of data between different subroutines through global variables, such as the continuous variables in Fortran’s MODULE function. In this way, this information is available for both the main VUMAT and the DAMAGE subroutine.

The “DAMAGE” subroutine is called by VUMAT and has the function of updating the value of the damage parameter *D*. To do this, it uses a series of information contained in the global variables through the MODULE function, such as the connectivity map, coordinate map and the values of the local parameters $\lambda \prime_{e}$ and $\sigma_{eq}$ of all elements, to calculate the non-local average parameters of $\sigma_{eq}^{*}$ and $\lambda_{e}^{*}$. The use of global variables is necessary since this information from neighboring elements at the external surface is used in this model, and not only the information from the material element analyzed.

**Figure 4:**
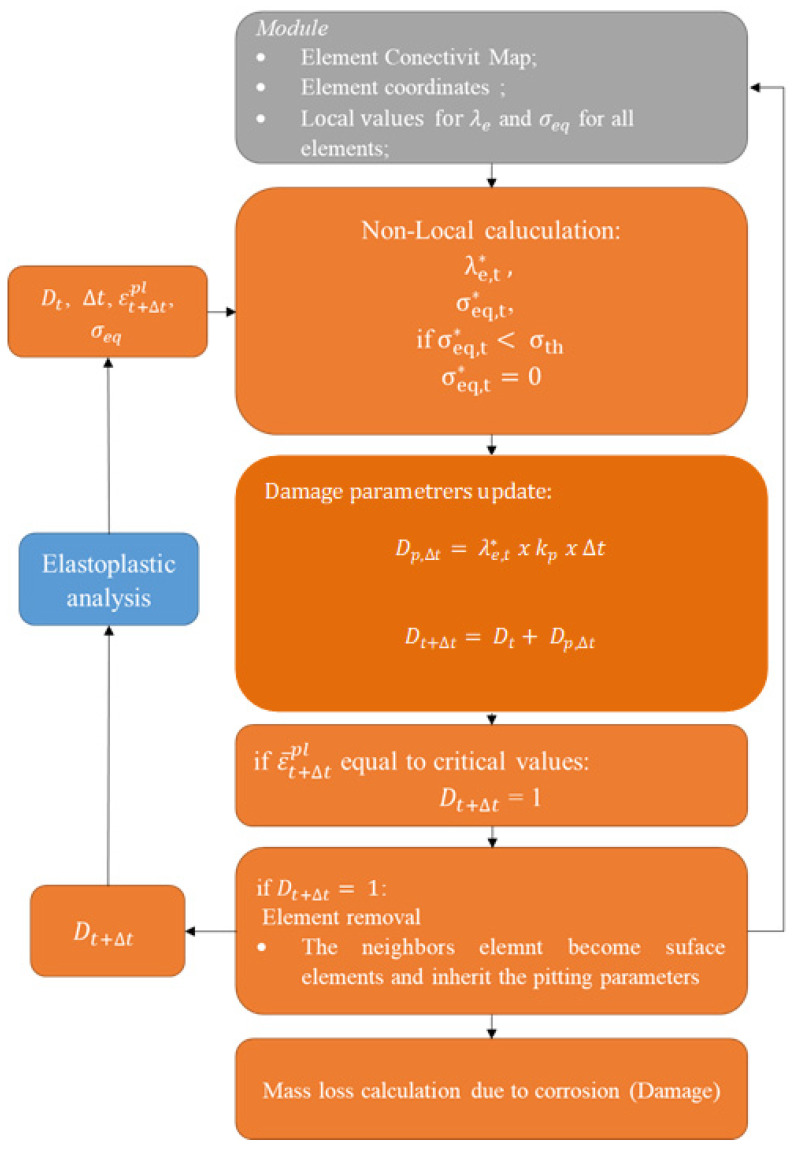
Operation flowchart of the “DAMAGE” subroutine.

When an element is completely corroded, or when the equivalent plastic strain ${\bar{\varepsilon }}^{pl}$ reaches a critical value, the subroutine removal of the element from the mesh is signaled to the Abaqus^®^ solver. After the removal is signaled, the DAMAGE function updates the model surface and triggers the mechanism for transferring the pitting parameter from the corroded element to its neighbors, according to Equation (7), thus, updating the MODULE global variables. In addition, after calculating the damage parameter, the subroutine performs the calculation of the element’s corroded mass percentage. Figure 4 presents a flowchart of the operation of the “DAMAGE” subroutine.

### 2.8. Simulation of In Vitro Biodegradation Experiments

To simulate the experiments used in the calibration of model parameters, a finite element model of the biodegradation specimens was created using the Abaqus^®^ software. The modeled geometry will have the same dimension of the specimen used in the in vitro biodegradation experiments and the same geometry for the uniaxial tensile tests.

**Figure 5:**
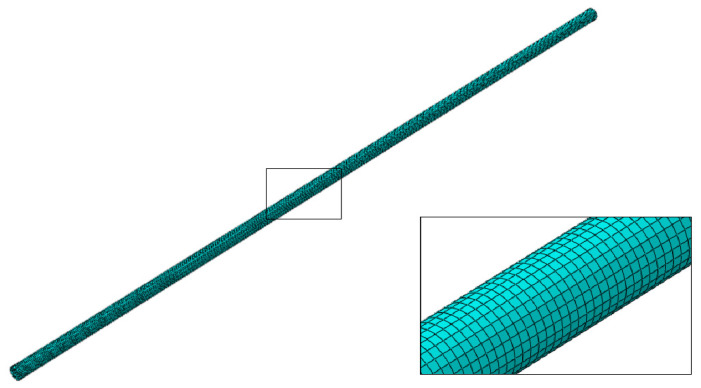
Detail of the mesh used in the representative geometry.

The simulation of biodegradation was carried out for different percentages of mass loss, and the chosen values of mass loss are associated with the values found in the experimental tests, where the effects of biodegradation on the mechanical properties were analyzed. In a second step, the uniaxial tensile test was simulated through the application of a controlled displacement to the top end of the specimen, while the bottom end was fixed. The displacement was incremented on the top end until failure. The load was calculated by adding the loads on all the elements at the top end surface. This way, it was possible to draw the load–displacement curve and compare it to the experimental result. A total of 104,244 8-node hexahedra-reduced integration solid elements (C3D8R) were used, having element sizes of 0.07 mm (Figure 5). The dimension of 0.07 mm is within the range of values commonly used in the literature for pitting corrosion simulation, which is related to the length of the corresponding geometry of corrosion regions in biodegradable magnesium alloys [12,22,23].

### 2.9. Calibration Strategy–Localized Corrosion Model

Concerning the mechanical behavior of the material, the elastoplastic model was adjusted based on the experimental results obtained in the mechanical characterization tests. Experimental tensile test results of corroded specimens from different degradation stages were considered.

To calibrate the localized corrosion model, the results obtained through the experimental tests were used to adjust the effective corrosion parameters: γ, ψ, β and $k_{p}$ to obtain a good correlation between the simulated behavior and what was observed experimentally.

Initially, the parameters *γ* and *β* were calibrated so that the model reflects the reduction in the mechanical integrity of the specimens induced by corrosion, as found in the experimental tests of the post-corrosion tensile test (described in the Section 2.5). This reduction is due to mass loss and the corresponding cross-sectional area which influences tensile, compressive, flexural and torque strengths. Specifically, a good correlation should be found for the tensile strength limit versus mass loss, and elongation versus mass loss curves. The adopted value of the scale parameter of the Weibull distribution, *ψ*, was 1 in all scenarios.

After adjusting parameters *γ* and *β*, parameter *k_p_* was calibrated so that the model reflected qualitatively (the trend) and quantitatively (average rate of biodegradation) the biodegradation behavior—mass loss vs. time curve—found for the alloy in the experimental immersion tests, as described in Section 2.4. The calibration of all parameters based on experimental results, as mentioned in this chapter, will be further discussed and presented in the following section (Results and Discussion).

Initially, a set of parameters was used as a reference for the iterative calibration process, in which the simulation results of a model are compared to experimental results. Aiming to decrease the difference between experimental and simulation results, at each iteration, the values for parameters *γ*, *β* and *k_p_* were based in the literature [4,16]. Therefore, the initial selection of parameters was restricted to values found in the literature that use similar methodologies for corrosion simulations of the magnesium alloy WE43.

### 2.10. Case Study–Orthopedic Screw Pull-Out Testing

The effectiveness of an orthopedic screw in assisting osteosynthesis directly depends on its fixation loads to the bone. These fixation constrains and loads applied to the surface are related to several characteristics of the screw, such as the diameter, diameter/core ratio, thread pitch, manufacturing material, and the substrate to which it was implanted [24]. Pull-out force is one of the most traditional metrics for measuring orthopedic screw fixation efficiency.

Therefore, a case study was proposed to analyze the influence of corrosion on the fixation strength of orthopedic screws. For this reason, a pull-out test was simulated for the screw in its intact state and at different corrosion levels, enabling the analysis of the pull-out strength variation for different degradation stages.

Pull-out tests are performed by inserting the threaded part of the screw into a substrate which can be made of bone tissue or synthetic materials, such as polyurethane. After insertion, the substrate is held in place, and an external displacement is applied to the screw until system failure occurs. Different system failure mechanisms may occur, depending on material properties, which may occur due to the screw failure or rupture of the insertion material substrate. The maximum force found in this test is taken as the force required for the screw pull-out.

A lag-cannulated screw, with an external diameter of Ø2.2 mm, commonly used in fractures of the extremities of the upper and lower limbs, was used as a reference.

**Figure 6:**
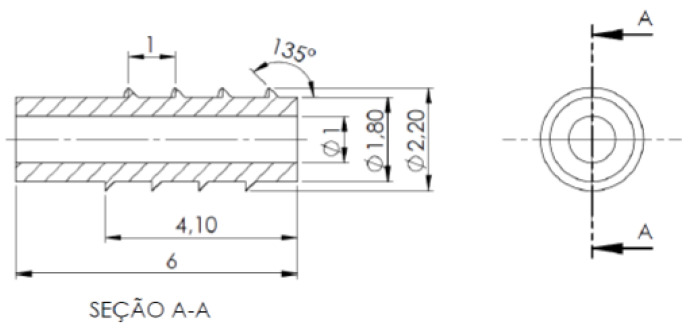
The main dimensions of the simulated orthopedic screw; dimensions in mm.

The WE43 magnesium alloy orthopedic screw was modeled according to the dimensions shown in Figure 6, and a mesh with elements of the linear hexahedral type with eight nodes (C3D8R) with dimensions of 0.07 mm was applied, resulting in a composite mesh of 41,844 elements.

**Figure 7:**
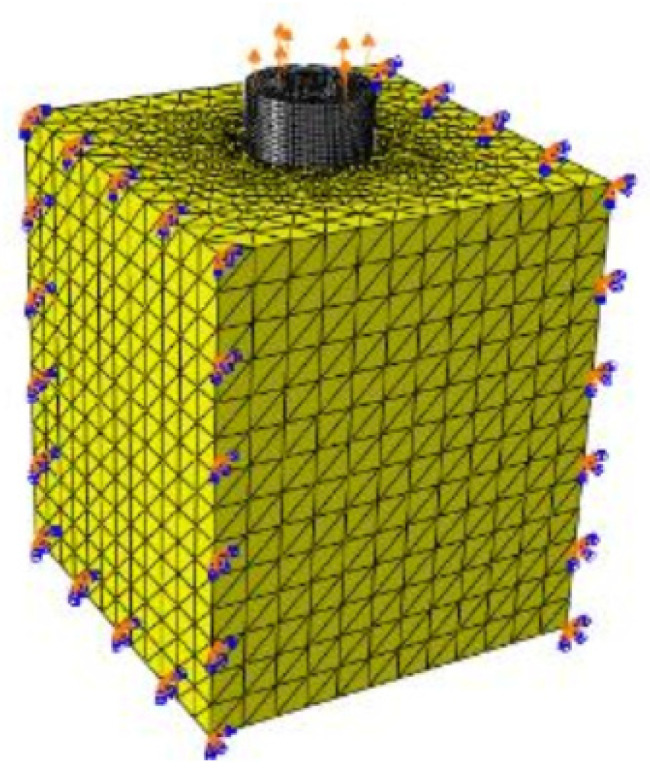
Boundary conditions of pull-out simulation.

The substrate, in turn, was considered to be entirely made up of trabecular bone and was modeled as a block measuring 6 mm by 6 mm by 10 mm. Regarding the mechanical behavior, the substrate was modeled as an elastoplastic material, coupled to a ductile damage mechanism, native to the Abaqus^®^ software, as proposed by Ketata et al. [25]. A total of 201164 10-node tetrahedra solid elements (C3D10M) were used for this block. The model considers hard contact for normal behavior and the tangential behavior was modeled with a penalty of 0.3 for the friction coefficient (Figure 7).

## 3. Results

### 3.1. Mechanical Characterization Tests

**Figure 8:**
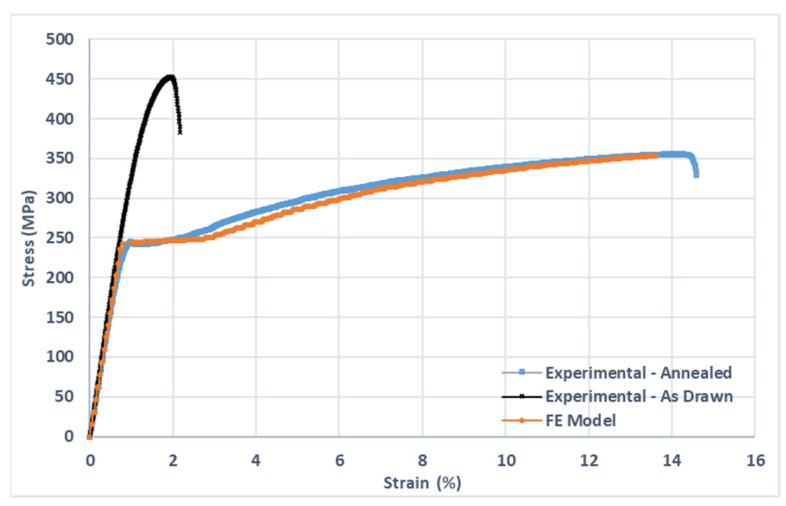
Average stress–strain curves from tensile testing of wire specimens, for both “As drawn” and “Annealed” conditions. The FE model results are also shown for comparison purposes.

Mechanical tensile test results are shown in Figure 8 in the form of stress–strain curves for both as-drawn and annealed conditions, and against the simulated results for the annealed condition. In Table 1, the same results are presented in the form of average values for modulus of elasticity, yield strength, ultimate tensile strength (UTS) and elongation at break, for both as-drawn and annealed conditions. The material was characterized after thermal treatment of homogenization.

As can be seen from Figure 8, the annealing process has a large impact on the mechanical properties of the WE43 alloy. An increase in 5.6 times was noted for the elongation at break, with 21.5% of reduction in UTS. The results of the mechanical characterization test are summarized in Table 1. The results of uniaxial tensile tests for both as-drawn and annealed WE43 alloy wires are in accordance with the findings of Griebel et al. [26] and Maier et al. [18] in similar material geometry and thermomechanical processing conditions [18,26]. Different values for UTS and elongation at break have been reported for different specimen geometries, metallurgical conditions and thermal treatment, suggesting that the WE43 alloy mechanical properties can be largely tuned for different applications [23,27].

However, the improved toughness and a good compromise between yield strength and ductility of the annealed WE43 alloy is considered of great importance during the design and material selection [28]. The annealed condition presented a better compromise between mechanical strength, toughness and ductility, and is adopted as the thermomechanical condition for further experiments and simulations. Thus, the as-drawn condition was no longer used.

**Figure 9:**
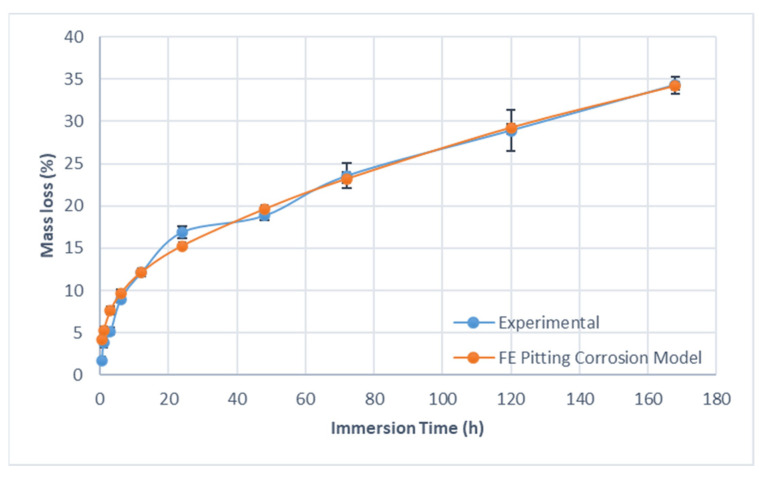
Mass loss versus corrosion time from corrosion experiment 1. The FE pitting corrosion model results are also shown for comparison purposes. Error bars represent a single standard deviation from the mean (n = 3).

The mechanical behavior of the magnesium alloy WE43, in its intact state, that is, without corrosion, was modeled using the von Mises elastoplastic model with isotropic hardening. The results of the mechanical characterization test, summarized in Table 1, were used to identify the plastic hardening behavior of the material. Specifically, the values of 245.9 MPa, 31.5 GPa and 0.27 were used as the initial parameters of yield stress, elastic modulus, and Poisson’s ratio, respectively. Figure 9 presents a comparative stress–strain curve between the results obtained experimentally and the simulated result. It is possible to note that the FE model was able to properly simulate the elastic–plastic behavior of the annealed WE43 magnesium alloy.

### 3.2. Determining the Biodegradation Rate

Through immersion experiments in SBF, using the hydrogen evolution technique, it was possible to calculate the in vitro biodegradation rate of the studied alloy for 7 days.

Figure 9 shows the mass loss of the specimens after different immersion periods, obtained from the hydrogen evolution test, totaling an average mass loss of 34.3% after 168 h of immersion, in which it was verified that 50% of the total biodegradation occurred in the first 24 h of the test. It is possible to verify that the biodegradation rate has a variable behavior over time. A high rate of biodegradation is observed in the first 6 h of immersion, with a sharp decrease in the later periods, resulting in an average total biodegradation rate of 0.080 mg cm^2^ h^−1^. The variable corrosion rate is in accordance with the findings of Ascencio et al. [29] and may be explained by the formation of an oxide layer on the material surface, which slows down the corrosion process [29,30].

**Figure 10:**
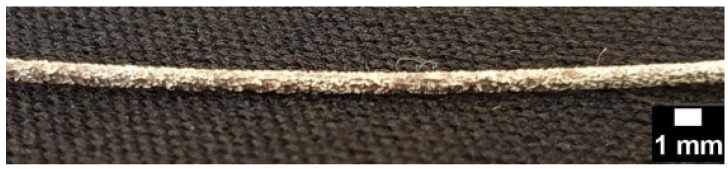
Specimen after 168 h corrosion.

Figure 10 shows an image of the specimen after 168 h corrosion, in which it is possible to observe the inhomogeneous corrosion presented by the magnesium alloy WE43.

Initially, corrosion occurs preferentially in certain regions of the material’s microstructure that act as a local cathode, such as grain boundaries and impurities, and in surface regions where the natural passive oxide layer is broken, resulting locally in a higher corrosion rate [29]. With the evolution of the corrosion process, the formation of magnesium oxides from the corrosive process itself occurs on the surface of the material, reducing the contact area between the electrolyte and the substrate, thus, resulting in the deceleration of degradation.

Quantitatively, the average corrosion rate was experimentally determined and is within the range reported in the literature, ranging from 0.011 mg ${\mathrm{cm}}^{2}h^{-1}$ to 0.89875 mg ${\mathrm{cm}}^{2}h^{-1}$ [23,31]. Such variation can be explained by the high influence of the geometry of the specimen, or the surface finish and the electrolyte used on the biodegradation rate. Based on these results, further investigations of these variations should be carried out in future studies.

By combining the results of in vitro biodegradation tests and the post-biodegradation tensile tests, it was possible to define the influence of corrosion on the mechanical strength of the material.

**Figure 11:**
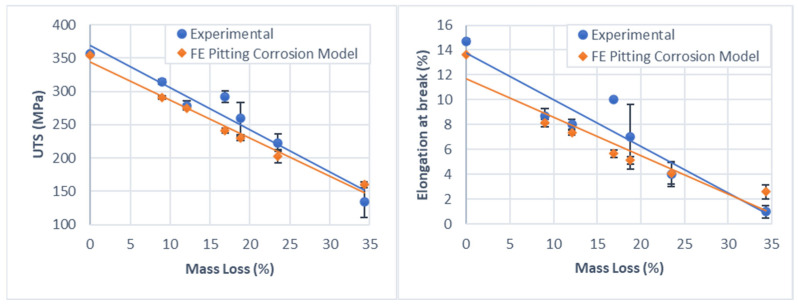
Reduction in material UTS (MPA) and elongation at break (%) due to degradation, based on the results of experiment 2. The FE pitting corrosion model results are also shown as comparison. Error bars represent a single standard deviation from the mean (n = 3).

In Figure 11, it is possible to notice that an increase in the percentage of corroded mass results in a reduction both in the mechanical strength, represented by the UTS, and in the elongation at break. The correlation between the percentage of mass loss and both properties showed a linear evolution, with R^2^ = 0.95 and 0.89 for the UTS and the elongation at break, respectively. For relatively low percentages of mass loss, 9%, the UTS is reduced by 12.0% and the elongation at break by 40.8%. The reduction in mechanical strength is due to a decrease in the cross-sectional area while the material is being corroded and the elements on the numerical model are being removed. For higher percentages of corrosion, around 34.5% of mass loss, these values rise to 62.2% and 93.2%, respectively. Furthermore, by performing a graphical extrapolation, it is possible to stipulate that corrosion between 55% and 60% would result in a complete loss of mechanical strength of the specimen. The linear reduction on mechanical properties due to corrosion has also been reported by other authors [18,31].

### 3.3. Localized Corrosion Model

**Figure 12:**
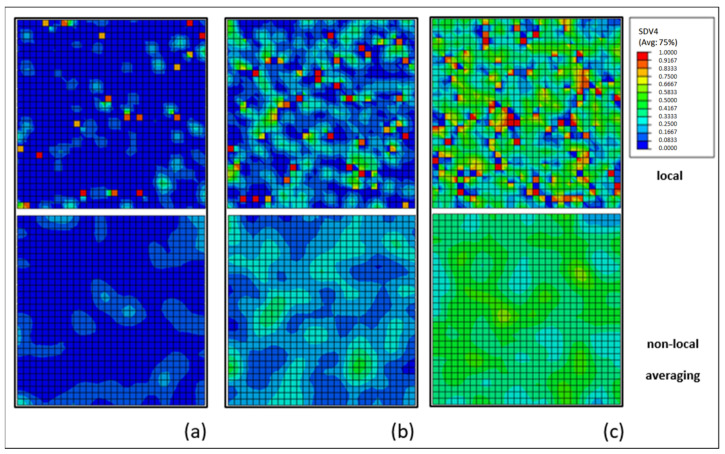
Influence of different values for *γ* on the distribution of local pitting parameters, $\lambda_{e}$, (upper part) and the effect of application of the non-local averaging method (lower part); (**a**) *γ* = 0.1, (**b**) *γ* = 0.4 and (**c**) *γ* = 0.9.

The pitting corrosion model was successfully implemented and calibrated based on the results of biodegradation experiments. The iterative calibration procedure required two calibration stages. In the first stage, the calibration of parameters *γ* and *β* was based on the reduction in mechanical properties due to corrosion. As discussed before, the dimensionless parameter *γ* is related to the Weibull distribution and controls the uniformity of the corrosion process on material surface. Figure 12 illustrates the difference in the distribution of local pitting parameter, $\lambda_{e}$, for different values of *γ*. High values of *γ* lead to faster and more homogenous corrosion. The non-local averaging method for the pitting parameter has a huge impact on pitting distribution, as can be seen in Figure 12. The dimensionless parameter *β* controls the pitting penetration rate within the specimen, where lower values for *β* lead to a more superficial corrosion patterner.

In the second calibration stage, the calibration of parameter $k_{p}$, for the chosen set of parameters *γ* and *β*, was based on the corrosion trend and corrosion rate observed experimentally by the hydrogen method. The kinetic parameter $k_{p}$ is intrinsically related to the corrosion rate. While in the literature, all magnesium pitting corrosion models available adopt a constant corrosion kinetic parameter, the present work introduced a variable corrosion kinetic parameter as a function of corrosion time, hence, improving the simulation results by enabling the representation of the variable nature of the corrosion rate of different magnesium alloys. The intrinsic length *Lr* was adopted as 0.2 mm in all scenarios, according to Ma et al. [22].

The calibrated parameters for the corrosion pitting model are presented on Table 2. After the final calibrated model parameters were set, each simulation was run two more times, with different seeding used in the Weibull distribution step, resulting in the addition of two different corrosion results. The final simulation results presented in Figure 10 and Figure 12 are the mean and the standard deviation values of these three simulations.

As can be seen from Figure 9, the calibrated pitting corrosion model can accurately represent the variable corrosion rate and provide the same average corrosion rate found experimentally; it was only possible due to the introduction of a variable corrosion kinetic parameter, as discussed before. Additionally, from Figure 11, it is possible to note that the pitting corrosion model can accurately capture the linear reduction in material structural integrity, represented in the form of the UTS and elongation at break, observed experimentally.

The introduction of a variable corrosion kinetic parameter on the pitting corrosion model adds extra level of possibilities regarding the fit of mass loss due to corrosion plots. The difference in parameter values found in this work and reported in the literature for the WE43 magnesium alloy is an indication that the model must be recalibrated for different material and testing conditions, which is a limitation of the phenomenological approach [22,23]. Although in vitro and in vivo corrosion rates vary greatly, most data in this work are presented regarding the function of mass loss instead of immersion time, which is a better characterization of the specimen structural integrity for a given situation, and may help with future data extrapolation.

**Figure 13:**
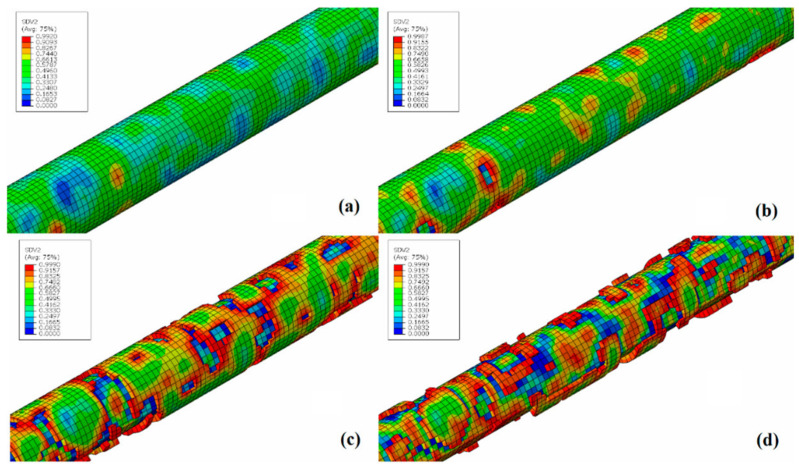
CP geometry during localized corrosion simulations for different immersion times. (**a**) = 24 h, (**b**) 48 h, (**c**) 96 h and (**d**) 168 h.

**Figure 14:**
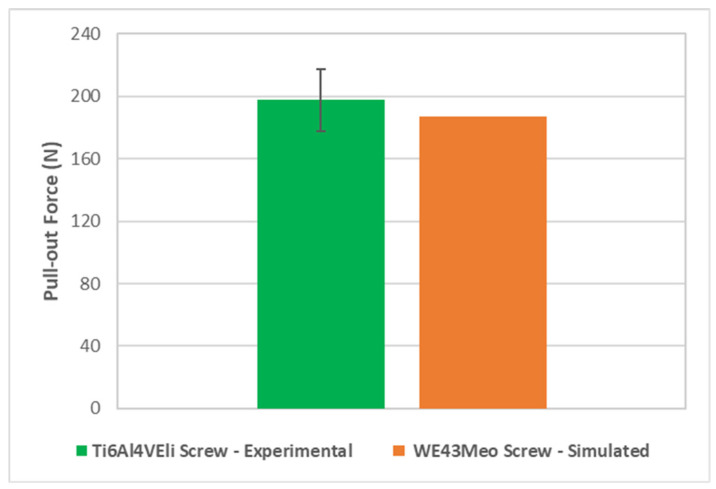
Comparative pull-out strength—experimental results for a screw made of titanium alloy Ti6Al4VEli and the simulated results. In the case of experimental data, n = 5.

Figure 13 shows the specimen used in the calibration simulations at different stages of the corrosion process, corresponding to an approximate immersion time of 24 h, 48 h, 96 h and 168 h. It is possible to notice, through Figure 14, that the model was able to reproduce the localized corrosion mechanism characteristic of the magnesium alloy WE43, as observed in vitro biodegradation tests and as reported in the literature by different authors, in which different corrosion pits nucleate randomly, grow and unite [23,30].

### 3.4. Case Study–Orthopedic Screw Pull-Out Testing

Initially, to validate the bone tissue model used for the substrate and the boundary conditions of the pull-out test, the pull-out force found for the simulated screw in its intact state was compared to the pull-out force found for an orthopedic screw manufactured in titanium alloy Ti6Al4VEli of similar geometry, supplied by Razek Equipamentos (São Carlos, Brazil), and was tested following ASTM F5434 [32]. It is possible to notice in Figure 14 that there is a good degree of correspondence between the simulation result and the experimental test result.

It is possible to notice in Figure 14 that there is a good agreement between simulation and experimental test results. Polyurethane 40 PFC was used as a substrate in these experimental tests since it has very similar mechanical characteristics to those of trabecular bone [33]. Furthermore, the screw geometry, as well as the insertion depth used in the specimen, are identical to those used in experimental tests. According to Chapman et al. [34], these are predominant factors for the pull-out test results, especially in cases such as the one in the present work, where failure preferentially occurs on the substrate, instead of on the implantable screw [34].

**Figure 15:**
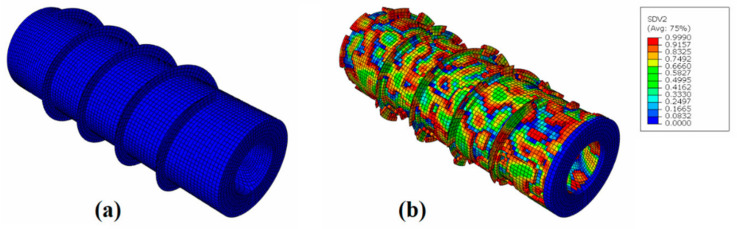
(**a**) Simulated screw in its intact state and (**b**) after 34.3% mass loss.

After adjusting the boundary conditions and performing the simulation for the solid screw, the pull-out test was simulated for the screw with different levels of degradation: 10%, 20% and 34.3% of mass loss. Figure 15 shows the orthopedic screw in its intact state and after 168 h of corrosion, equivalent to a mass loss of 34.3%.

**Figure 16:**
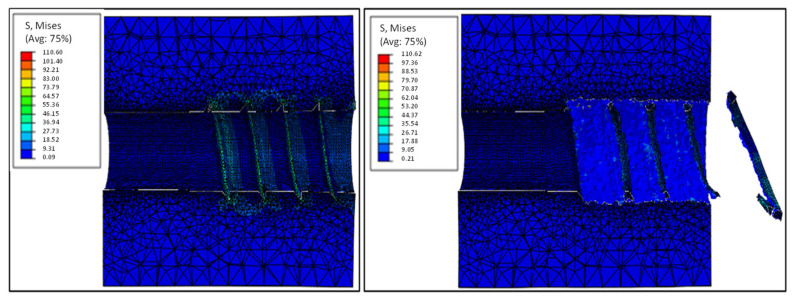
Stress distribution in the pull-out test substrate.

Figure 16 shows the stress field in the insertion substrate for two different moments of the test: close to the maximum load level and at the end of the test. It is possible to notice that the elastoplastic model, coupled to the ductile damage model, allows the removal of the mesh elements, thus, simulating the failure on the substrate.

**Figure 17:**
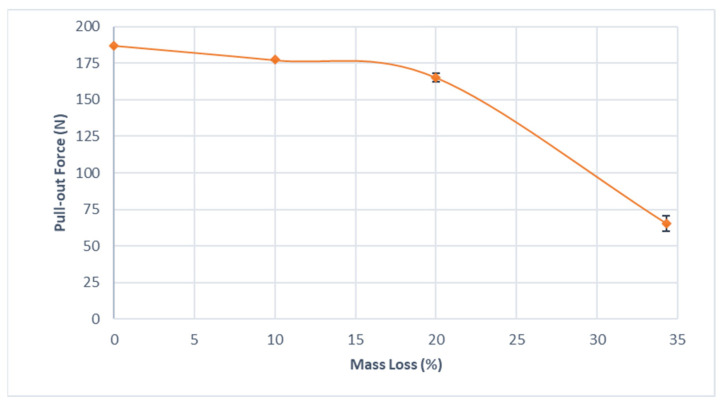
Pull-out force vs. mass loss. The error bars represent the standard deviation from the mean (n = 3).

Figure 17 presents the results of the pull-out tests in the form of pull-out vs. mass loss. Each scenario was simulated three times with different random initial seeds being provided by the Weibull distribution, resulting in three distinct distributions of the initial values of the pits.

The reduction in pull-out force showed a non-linear character, in which the screw can maintain 88% of the original pull-out force after 20% of mass loss. However, after a mass loss of 34.3%, the screw offers only 35% of the holding capacity of the intact screw.

According to Ilyas, Mahoney and Bucklen [35], the compressive force involved in fracture fixation when it is using Ø2.4 mm orthopedic screws, equivalent to the screw object of the present case study, is of the order of 65 to 70 N; thus, it is assumed that the screw is able to sustain the loads necessary for the correct stabilization of fractures until a degradation of approximately 30% to 35% of mass loss [22]. However, it is important to emphasize that the model in question does not take into account the regeneration and adhesion of bone tissue in the regions adjacent to the orthopedic screw, which could result in changes in the pull-out forces found in vivo.

## 4. Conclusions

In the present work, a finite element corrosion model, based on the continuum damage mechanics framework, was successfully implemented and calibrated based on experimental data to simulate the influence of corrosion on the structural integrity of biodegradable magnesium alloys. This phenomenological model allows to simulate the main magnesium corrosion mechanism: pitting corrosion. Unlike other methodologies found in the literature, the model implemented in this work introduces a variable corrosion kinetic parameter. The non-local pitting corrosion model was calibrated based on experimental data collected from in vitro corrosion experiments and the mechanical testing of magnesium WE43 alloy wire specimens. The calibrated pitting corrosion model accurately captures both the linear mechanical reduction in specimen strength, in the form of ultimate tensile stress and elongation at break with mass loss, as well as the non-linear mass loss of magnesium WE43, observed experimentally. The corrosion model was applied to a case study involving the pull-out test of a magnesium orthopedic screw and was able to capture the expected loss of screw pull-out force due to corrosion. The proposed numerical model proved to be an efficient tool in the evaluation of the structural integrity of biodegradable magnesium alloys and bone-implant assembly. This methodology can be used in future works for the design optimization and pre-validation of orthopedic implants.

## Figures and Tables

**Table 1:** Average mechanical properties for both as-drawn and annealed wire specimens (n = 3).

Thermal Condition	Modulus of Elasticity (GPa)	UTS (MPa)	Yield Stress (MPa)	Elongation at Break (%)
**As-Drawn**	35.6 ± 2.0	454.4 ± 8.3	394.9 ± 10.2	2.20 ± 0.02
**Annealed**	31.5 ± 0.6	356.7 ± 2.4	245.9 ± 8.5	14.7 ± 0.2

**Table 2:** Summary of localized corrosion model parameters.

*γ*	*ψ*	*β*	*k_pv_*
0.4	1	0.8	0.3125*t*^−0.68^

## Data Availability

Not applicable.
